# Measuring EQ-5D-5L utility values in parents who have experienced perinatal death

**DOI:** 10.1007/s10198-024-01677-z

**Published:** 2024-02-25

**Authors:** Elizabeth M. Camacho, Katherine J. Gold, Margaret Murphy, Claire Storey, Alexander E. P. Heazell

**Affiliations:** 1 Institute of Population Health, University of Liverpool, Liverpool, UK; 2 School of Health Sciences, University of Manchester, Manchester, UK; 3 Department of Family Medicine, University of Michigan Medical School, Ann Arbor, MI USA; 4 School of Nursing and Midwifery, University College Cork, Cork, Ireland; 5 Tommy’s Stillbirth Research Centre, University of Manchester, Manchester, UK; 6 Maternal and Fetal Health Research Centre, School of Medical Sciences, University of Manchester, Manchester, UK; 7 Saint Mary’s Hospital, Manchester University NHS Foundation Trust, Manchester, UK

**Keywords:** Health utility values, EQ-5D, Stillbirth, Neonatal death, Foetal death, I1, I19

## Abstract

**Background:**

Policymakers use clinical and cost-effectiveness evidence to support decisions about health service commissioning. In England, the National Institute for Health and Care Excellence (NICE) recommend that in cost-effectiveness analyses “effectiveness” is measured as quality-adjusted life years (QALYs), derived from health utility values. The impact of perinatal death (stillbirth/neonatal death) on parents’ health utility is currently unknown. This knowledge would improve the robustness of cost-effectiveness evidence for policymakers.

**Objective:**

This study aimed to estimate the impact of perinatal death on parents’ health utility.

**Methods:**

An online survey conducted with mothers and fathers in England who experienced a perinatal death. Participants reported how long ago their baby died and whether they/their partner subsequently became pregnant again. They were asked to rate their health on the EQ-5D-5L instrument (generic health measure). EQ-5D-5L responses were used to calculate health utility values. These were compared with age-matched values for the general population to estimate a utility shortfall (i.e. health loss) associated with perinatal death.

**Results:**

There were 256 survey respondents with a median age of 40 years (IQR 26–40). Median time since death was 27 months (IQR 8–71). The mean utility value of the sample was 0.774 (95% CI 0.752–0.796). Utility values in the sample were 13% lower than general population values (*p* < 0.05). Over 10 years, this equated to a loss of 1.1 QALYs. This reduction in health utility was driven by anxiety and depression.

**Conclusions:**

Perinatal death has important and long-lasting health impacts on parents. Mental health support following perinatal bereavement is especially important.

**Supplementary Information:**

The online version contains supplementary material available at 10.1007/s10198-024-01677-z.

## Introduction

Healthcare funding is limited. In state-funded healthcare systems which aim to provide health benefit across the population, it is important that interventions should provide clinical benefit for an acceptable economic investment. In England, Wales, and Northern Ireland, the National Institute of Health and Care Excellence (NICE) recommends what the National Health Service (NHS) should fund, their decisions are informed by research evidence about how effective and cost-effective different treatments and services are. NICE use a generic threshold to judge if an intervention is cost effective; if the additional cost of an intervention is less than £20,000 to improve health by one quality-adjusted life year (QALY), it is considered potentially cost effective [1]. This threshold is applied to interventions that may improve health, prolong life, or both and so QALYs are used as the measure of health benefit because they combine quality (i.e. utility values) and quantity of life into a single number. Decision-makers in other countries also use a formal threshold in terms of cost per QALY to assess cost-effectiveness of health interventions including Ireland, Thailand, Poland, and Sweden, and a number of other countries use an informal cost per QALY threshold [2].

In 2021 there were 624,828 live births in England and Wales and there were 3950 perinatal deaths (2597 stillbirths and 1353 neonatal), a rate of 6.3 per 1000 live births [3]. There is a paucity of research regarding the health economic impact of perinatal death (when a baby is stillborn or dies shortly after birth). A recent systematic review identified four studies that identified the direct costs of a stillbirth (i.e. of providing care for the birth and investigations to determine the cause of death) ranged from $6934 to $9220 (USD; 2020) per baby stillborn [4]. Critically, the review acknowledged that 97% of costs are likely to be indirect (e.g. time off work). It is likely that intangible costs, defined as non-monetary costs reflecting the “disvalue” to an individual of pain, anxiety, fear, and suffering, are much greater than direct and indirect costs combined [5]. Synthesis of the psychological outcomes following stillbirth identifies that negative symptoms such as anxiety, depression, chronic pain and fatigue, and post-traumatic stress disorder are frequently reported [6]. Thus, knowledge of direct, indirect, and intangible costs of perinatal death are important to accurately estimate the size of the impact on families and health services and to inform policy- and decision-making.

To date there have been no studies that have assessed quality of life (in terms of shortfall in utility values and QALYs) to measure the impact of perinatal death. The challenges in doing so have been highlighted by Philips and Millum who argue that the impact of the death of a baby should be assessed using utility values but acknowledge that the estimate would vary significantly (up-to > 30-fold difference) depending on whether the impact is calculated from solely the effects perceived by the mother or whether the loss of life of the baby is considered [7, 8]. The lack of information about utility values following perinatal death means that currently cost-effectiveness evidence in maternity care is less robust than other clinical disciplines. This project aimed to estimate the health impact of perinatal death on parents to calculate utility values which can be used in future economic evaluations.

## Methods

### Study design, setting, and participants

This was an opportunistic cross-sectional study, collecting online survey data from participants at a single time point. Parents living in the United Kingdom (UK) who had ever experienced a perinatal death (stillbirth or neonatal death) were invited to participate in this study. The sample size was determined by pragmatic considerations (i.e. as many participants as it was possible to recruit during the period in which the survey was open) rather than a formal sample size calculation.

All procedures involving human participants were approved by the University of Manchester Research Ethics Committee (Reference: 2022-12934-25770). Informed consent was obtained from all participants prior to completing the online survey.

The survey was hosted online using the Qualtrics XM platform (Qualtrics, Provo, UT, USA). It was publicised using social media. Data were collected between 6 April 2022 and 31 December 2022. The survey included questions about the parents’ age (collected in 5-year age bands with a lower bound of < 20 years and an upper bound of 75 + years) and birthing role (mother/birthing parent or father/partner), the month and year of any stillbirths/neonatal deaths they experienced, and the month and year of any pregnancies following perinatal death. Information that could identify individual participants during or after data collection was not collected. We did not collect any other data on participant characteristics to ensure that participants felt comfortable with the level of disclosure, to provide reassure that they could not be identified from their responses, and to minimise burden on them. The time since most recent perinatal death was calculated in months for each participant. Septiles were used to identify subgroups within the sample, based on time since loss. We used the septile boundaries to identify groupings of time since loss that would be useful for other analysts. These were as follows: 0–6 months, 7–12 months, 13–24 months, 25–36 months, 37–60 months, 61–120 months, and ≥ 121 months.

### Outcome measures

Participants were asked to complete the EQ-5D-5L instrument [9]. This is a generic measure of health status that asks participants to rate their health across five domains (mobility, self-care, usual activities, pain/discomfort, anxiety/depression) on a scale from 1 (no problems on that domain) to 5 (severe problems on that domain). Participants also rated their health on a visual analogue scale (0 = worst health; 100 = best health). Responses on the 5 domains were translated into health utility values (0 = dead; 1 = perfect health) using a mapping algorithm from an English value set for the EQ-5D-3L as recommended by NICE at the time of analysis [10, 11]. The algorithm assigns different utility values for male and female respondents. We did not ask respondents to report their sex and so for the derivation of utility values it was necessary to assume that mothers/birthing parents were female and that fathers/partners were male. The algorithm assigns different utility values based on age according to the following age bands: 18–34, 35–44, 45–54, 55–64, and 65 + years.

### Comparison to the general population

Our goal was to estimate the utility shortfall for the sample compared to the general population in England. If everyone uses the same general population benchmark for calculating utility shortfalls for different subgroups, this enhances methodological standardisation and comparability between studies by policymakers. The NICE Decision Support Unit recommend a specific set of general population utility values for England [12]. The Decision Support Unit used data from a large nationally representative survey conducted in England (The Household Survey for England, 2014) to estimate general population utility values by age and sex [12]. Their estimates are adjusted to account for clustering, stratification, and weighting (for selection, non-response, and population profile in the original sample) to maximise representativeness to the population in England. Methodological standardisation and comparability were key aims of this work, and so rather than collect additional survey data from people who have not experienced perinatal death, we based our comparison on the recommended general population sample. We used these general population estimates to assign to each participant in our sample a norm utility value, based on age and sex. The general population values are reported for each single year of age, whereas our survey collected age in bands; therefore, we used the single year value that corresponded to the mid-point of each age band. The observed utility values were compared with the general population norms to calculate the impact of perinatal death on utility values. This was calculated as absolute (norm value minus observed value) and proportional (observed value divided by norm value) shortfalls in utility [12].

### Statistical analysis

Data analysis was conducted using STATA version 15 (StataCorp, College Station, TX, USA). Descriptive statistics are presented as means or medians (depending on the characteristics of the data) and proportions for categorical variables. EQ-5D-5L responses were summarised graphically. T-tests were used to compare population norm and observed utility values. This was done for the whole sample and by subgroups of the sample based on time since most recent perinatal death. This was also done for the subgroup of mothers/birthing parents (i.e. excluding father/partners from the analysis). A linear regression model was used to estimate the utility decrement with adjustment for whether or not participants (or their partners) had a subsequent pregnancy. We have reported 95% confidence intervals to reflect the statistical power achieved and the degree of uncertainty in the results. To minimise the impact of outliers (e.g. two participants whose baby died over 40 years ago), regression analysis was restricted to participants whose baby died within the last 10 years. The regression analysis included data for all of these participants together and then the results of the model were used to predict (using post-estimation commands in STATA) the utility decrements for subgroups defined by time since last perinatal death. The utility shortfall within each subgroup was combined to derive a QALY loss over the respective period of time. QALYs are calculated by dividing a utility value (or utility shortfall) by the period of time (measured in years) to which the utility value relates. For example, to calculate the 60-month (5-year) QALY loss we divided the mean utility shortfall for participants whose baby died in the last 5 years by 5. We have provided an illustrative example of the QALY loss calculation in Supplementary Material (Box 1). Total (cumulative) QALY losses over 12 months (1 year), 60 months (5 years), and 120 months (10 years) were estimated by combining the QALY losses of all relevant subgroups. For example, to estimate the QALY loss over 12 months, the loss over 0–6 months and 7–12 months were added together. The analysis was repeated for the subgroup of mothers/birthing parents only. In addition to our primary estimate which does not incorporate discounting of QALYs over time, total QALY losses over 60 and 120 months were also calculated at annual discount rates of 2.5%, 3.5%, and 5% for QALYs accrued beyond 12 months.

## Results

The survey was completed by 256 bereaved parents, 93% of whom were mothers/birthing parents. The characteristics of the sample are summarised in Table 1. The majority (87%) of the sample were aged between 18 and 44 years. Within the sample, the amount of time since respondents had experienced perinatal death varied widely, from a few months to over 40 years. The median time was 27 months. Almost three-quarters of the sample had had at least one subsequent pregnancy following perinatal death.

**Table 1:** Sample characteristics at time of survey completion

Characteristic	*n*/*N* (%) or median (IQR)
Age (years)	40 (26–40)
*Age group*	
18–34	112/256 (44)
35–44	110/256 (43)
45–54	26/256 (10)
55–64	6/256 (2)
65 +	2/256 (1)
Mother/birthing parent	238/256 (93)
Time since last perinatal death (months); *n* = 247	27 (8–71)
*Time since last perinatal death*	
0–6 months	43/247 (18)
7–12 months	38/247 (15)
13–24 months	37/247 (15)
25–36 months	25/247 (10)
37–60 months	32/247 (13)
61–120 months	38/247 (15)
121 + months	34/247 (14)
Had subsequent pregnancy	188/256 (73)
Time to subsequent pregnancy (months); *n* = 178	60 (36–84)

**Fig. 1:**
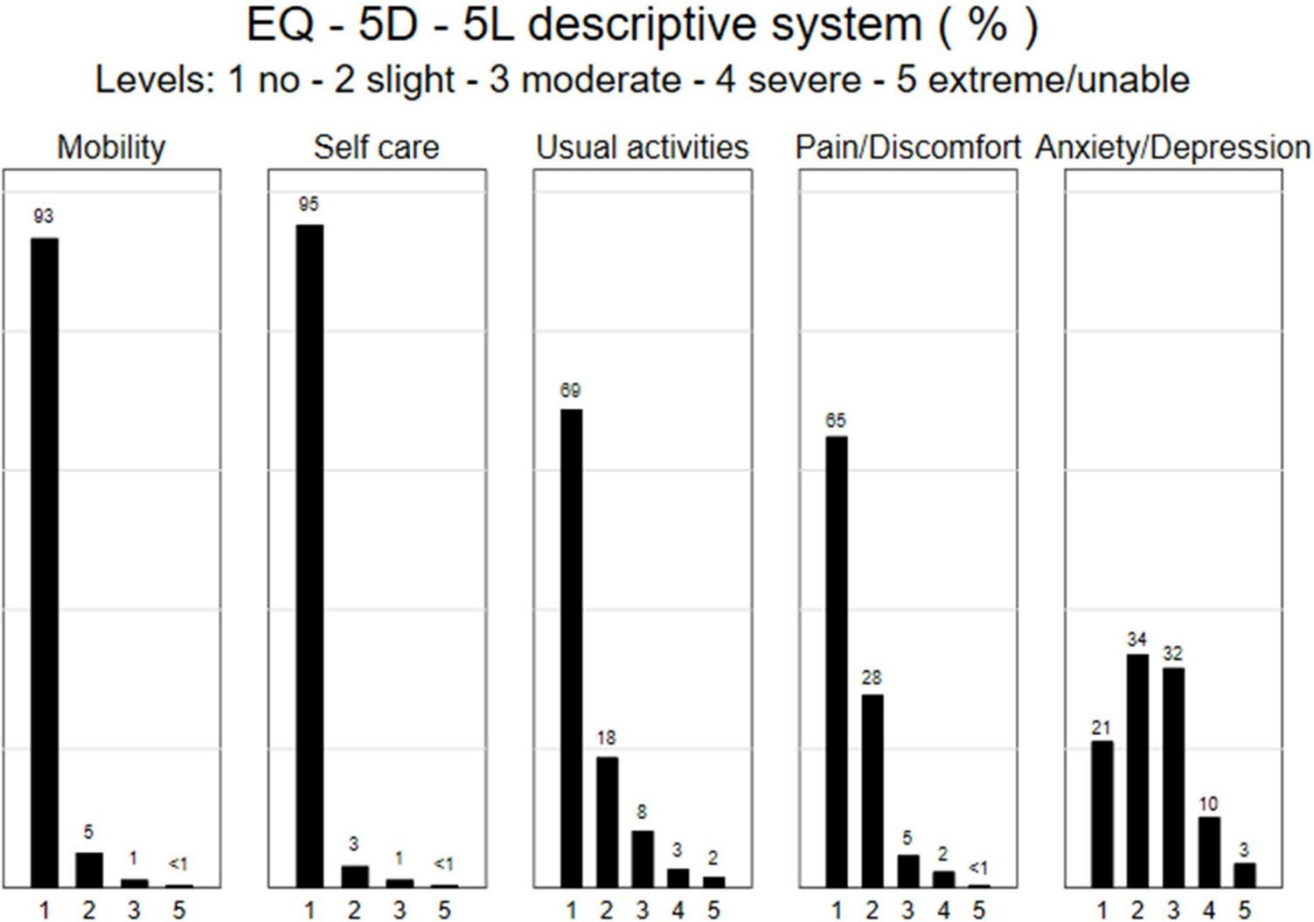
Distribution of responses on EQ-5D-5L

A summary of responses on the EQ-5D-5L is presented in Table 2. The majority of the sample (79%) reported at least some problems on the anxiety/depression domain of the EQ-5D-5L. The profile of responses across all domains is shown in Fig. 1. The mean utility value for the sample was 0.774 (95% CI 0.752–0.796), notably higher among fathers/partners than mothers/birthing parents (0.856 vs 0.767; *p* = 0.04), which is also reflected in the mean utility shortfall of the sample compared with the general population (13.8% in mothers/birthing parents versus 5.9% in fathers/ partners).

**Table 2:** EQ-5D-5L summary statistics

	*n*/*N* (%) or mean (95% CI)		
	Whole sample (*n* = 256)	Mother/birthing parent (*n* = 238)	Father/partner (*n* = 18)
No problems: mobility	239/256 (93)	221/238 (93)	18/18 (100)
No problems: self-care	244/256 (95)	226/238 (95)	18/18 (100)
No problems: usual activities	176/256 (69)	159/238 (67)	17/18 (94)
No problems: pain	166/256 (65)	151/238 (63)	15/18 (83)
No problems: anxiety/depression	54/256 (21)	59/238 (21)	5/18 (28)
EQ-5D-5L utility index value	0.774 (0.752–0.796)	0.767 (0.744–0.791)	0.856 (0.803–0.909)
EQ-5D visual analogue scale	69 (67–71)	69 (66–71)	74 (68–79)
Utility index value for general population (age- and sex-matched)	0.892 (0.890–0.895)	0.891 (0.888–0.894)	0.911 (0.900–0.922)
Absolute shortfall compared with general population utility index value	– 0.119 (– 0.141 to – 0.096)	– 0.123 (– 0.147 to – 0.100)	– 0.055 (– 0.112 to 0.002)
Proportional shortfall compared with general population utility index value	– 13.2% (– 15.7% to – 10.7%)	– 13.8% (– 16.4% to – 11.1%)	– 5.9% (– 12.1% to 0.2%)

Table 3 presents the mean utility values and mean proportional difference between observed and utility values and general population norm values. As observed utility values are lower than general population values, the difference is reported as “utility shortfall”. Utility values and shortfalls are reported for the whole sample, by time since perinatal death, and for the subgroup of mothers/birthing parents (i.e. excluding fathers/partners). Observed utility values are significantly lower than general population values, with a mean proportional shortfall of 13%. Utility values are lowest, and the utility shortfall highest in the period immediately following perinatal death. After the first 12 months, the utility shortfall begins to decrease, although this is not a straightforward linear relationship. Beyond 10 years after perinatal death, the shortfall is at its lowest (5%) and the difference is not statistically significant. As expected, there is little impact on the results of including only mothers/birthing parents in the analysis as they comprise the vast majority of the sample. There is very little difference in the proportional shortfall for the mother/birthing parent subgroup compared with the whole sample (less than 1% difference, 13.2% vs. 13.8%). Based on unadjusted mean values, parents who did not have another pregnancy following perinatal death had a greater utility shortfall than those who had another pregnancy. However, this does not account for the complex relationship between time since the death of their baby and opportunity for a subsequent pregnancy. Initially as the time increases so does the opportunity for a subsequent pregnancy to occur, although as mothers increase in age this opportunity decreases again.

**Table 3:** Unadjusted utility values and decrement, reported by time since most recent perinatal death at the time of completing the EQ-5D

	*n*	Utility values		Utility decrement		*p*-value for difference (sample vs general population)
		Mean	sd	Mean	sd	
Whole sample	256	0.7737	0.1795	– 13%	20	< 0.0001
*By time since most recent perinatal death*						
0–6 months	43	0.6683	0.1717	– 26%	19	< 0.0001
7–12 months	38	0.7317	0.1728	– 19%	19	< 0.0001
13–24 months	37	0.8243	0.1544	– 9%	17	0.004
25–36 months	25	0.7771	0.2588	– 14%	29	0.026
37–60 months	32	0.8126	0.1644	– 9%	18	0.008
61–120 months	38	0.7891	0.1597	– 11%	18	0.001
121 + months	34	0.8172	0.1357	– 5%	16	0.079
*Only mothers included*						
Mothers only	238	0.7675	0.1825	– 14%	21	< 0.0001
*Parents who had a subsequent pregnancy*						
Subsequent pregnancy	188	0.7924	0.1748	– 11%	20	< 0.0001
No subsequent pregnancy	68	0.7221	0.1835	– 20%	21	< 0.0001

Table 4 shows the utility shortfall and number of QALYs lost on average by a parent over the 1, 5, and 10 years following perinatal death. Results are shown for the whole sample and also for the subgroup of mothers/birthing parents in the sample. If the impact of perinatal death did not vary by time since the baby died we would expect that the QALY loss over the first year (12 months) would be approximately 10% of the total QALY loss over 10 years (120 months). In our sample, the QALY loss in the first 12 months accounts for 18% of the 10-year loss in the whole sample and in the subgroup of mothers/birthing parents. If the impact of perinatal death did not vary by time since death we would expect that the QALY loss over the first 5 years (60 months) was found to be approximately half (50%) of the total QALY loss over 10 years (120 months). The QALY loss over the first 5 years (60 months) accounts for 53% of the 10-year (120 months) QALY loss in the whole sample and 55% in the mothers/birthing parents subgroup. Together, this suggests that the greatest loss of health occurs sooner after the death of the baby but after an initial recovery after the first 12 months does not appear to improve any greater than this over the remainder of the 10-year period. The values reported in Table 4 are not discounted. The 0–60-month cumulative QALY loss at annual discount rates of 2.5%, 3.5%, and 5% is – 0.5562, – 0.5477, and – 0.5355. The 0–120-month cumulative QALY loss at annual discount rates of 2.5%, 3.5%, and 5% is – 0.9863 – 0.9498, and – 0.8995.

**Table 4:** Absolute utility shortfall and QALY losses over 12, 60, and 120 months following perinatal death

Time since most recent perinatal death	Whole sample (*n* = 213)		Mothers/birthing parents only (*n* = 199)	
	Utility shortfall mean (95% CI)	QALY loss	Utility shortfall mean (95% CI)	QALY loss
0–6 months	– 0.2216 (– 0.2856 to – 0.1576)	– 0.1108	– 0.2224 (– 0.2889 to – 0.1558)	– 0.1112
7–12 months	– 0.1660 (– 0.2247 to – 0.1074)	– 0.0830	– 0.1736 (– 0.2345 to – 0.1127)	– 0.0868
13–24 months	– 0.0791 (– 0.1375 to – 0.0207)	– 0.0791	– 0.0807 (– 0.1408 to – 0.0206)	– 0.0807
25–36 months	– 0.1289 (– 0.2025 to – 0.0552)	– 0.1289	– 0.1505 (– 0.2317 to – 0.0692)	– 0.1505
37–60 months	– 0.0886 (– 0.1537 to – 0.0235)	– 0.1772	– 0.0937 (– 0.1619 to – 0.0254)	– 0.1874
61–120 months	– 0.1022 (– 0.1619 to – 0.0424)	– 0.5110	– 0.0998 (– 0.1635 to – 0.0361)	– 0.4990

Cumulative QALY loss	Whole sample		Mothers only	
0–12 months	– 0.1938		– 0.1980	
0–60 months	– 0.5790		– 0.6166	
0–120 months	– 1.0900		– 1.1156	

Values are estimated from a regression model which was adjusted for whether or not participants had had a subsequent pregnancy following perinatal death. Participants whose baby died more than 10 years ago were excluded from the regression model.

Cumulative QALY loss is calculated by adding together QALY loss experienced in each period.

Note: values shown in table are rounded values whereas calculation based on non-rounded values

## Discussion

Parents who experience a perinatal death have significantly worse quality of life than the general population. The mean proportional utility shortfall is 13–14%, which over 10 years equates to a loss of around 1.1 QALYs (i.e. 1.1 years of perfect health). The prevalence of parents describing themselves as being anxious and/or depressed is a key factor contributing to lower health utility values. This is the first time that the QALY loss experienced by parents following perinatal death has been estimated.

Within our sample, parents who became pregnant again following the death of their baby had a significant utility shortfall compared with the general population. A subsequent pregnancy does not ameliorate the impact of perinatal loss; this is an important message from our analysis. Furthermore, subsequent pregnancies (and births) are likely to be distressing or traumatic and have been demonstrated to have an impact on the mental health of both mothers/birthing parents and fathers/ partners [13–17]. It is important that bereaved parents are provided with adequate and appropriate support through subsequent pregnancies. The parents in our sample who had not had a subsequent pregnancy since their baby died had a greater utility shortfall than those who had become pregnant again. The relationship between having a subsequent pregnancy and utility values observed in our sample is complex. For example, as the time since a baby died increases there is (theoretically) greater opportunity to become pregnant, whereas the utility shortfall is greatest in the first year after the baby has died. The utility shortfall does not decrease linearly beyond the first year after perinatal bereavement. This is likely to reflect that perinatal bereavement is not a process in which one “reduces grief over time” but rather moves between loss-oriented and restoration-oriented coping behaviours [18]; thus, it is likely that in the first year after loss there are more loss-oriented behaviours which move to restoration-oriented behaviour, but that the balance between these behaviours extends over the period studied here.

### Limitations

A key limitation of our analysis is that data were cross sectional. The relationship between time since baby death and health utility was explored using a snapshot of data from people at different stages after the death of their baby. The death of a baby affects parents in varied and long-lasting ways and may be felt some days more than others. Our study has not captured fluctuations over short or long periods of time. A longitudinal study which follows a cohort of people whose baby died would enable better understanding of this relationship. However, long-term cohort studies are not without their challenges including the costs to establish and maintain and loss to follow-up over time.

Our estimate for the 10-year QALY loss accounted for the relationship we observed between time since perinatal loss and utility shortfall (i.e. greater utility shortfall closer to the bereavement). However, it should be noted that within each of these subgroups there was a relatively small number of participants which contributes to uncertainty in our estimate. Others using our results in economic modelling should employ appropriate sensitivity analyses to account for this.

Our results appear to show that mental health (anxiety/depression) is a key driver of the utility shortfall. However, for mothers/birthing parents there is an association between experiencing repeated pregnancy loss/baby death and physical health conditions including autoimmune and cardiovascular diseases [19–22]. Thus, some of the utility shortfall we observed may be related to physical health conditions experienced by mothers/birthing parents. In our sample, 35% reported having pain or discomfort. This may reflect the presence of physical health conditions; however, a limitation of our work is that we did not collect data on specific health conditions as part of our survey. Some of the utility shortfall we observed in mothers/birthing parents may be related to recent pregnancy or childbirth [23] (33% of our sample had experienced baby loss in the previous 12 months). However, as the utility values from the general population came from a large, representative national sample, it is also likely that some respondents to that survey were or had recently been pregnant.

Another limitation is that we collected data on age and birthing role but not other sample characteristics (e.g. socioeconomic status, deprivation, immigration status, location within the UK, or race/ethnicity). Our justification for this was to limit the length of the survey (to minimise burden on participants) and limit the amount of personal and/or sensitive data collected so that participants were not concerned about being identified from their responses. However, this meant that we were unable to adjust our analysis for these factors. It also means that we are unsure how representative our sample is of the broader group of parents who have experienced perinatal loss. Another related limitation is that the algorithm for the derivation of utility values requires participants to be categorised as either “male” or “female”. Therefore, for the derivation of utility values it was necessary to make the assumption that mothers/birthing partners were female, and fathers/partners were male.

We used an English-language online survey to collect data which excludes people who do not read and understand English or have internet access. By advertising the survey on social media, and through third-sector organisations related to baby death, we are likely to have recruited people who are actively engaged with perinatal loss communities online. Especially when the loss occurred 20–30 years previous, this may represent those for whom the impact of the loss is most profound. As a result, we may have overestimated the impact of loss on parents’ health. However, we have limited our regression analysis to those whose baby/babies died within the last 10 years to minimise the impact of this on our results.

Finally, there are limitations of the EQ-5D which by design is a generic measure of health status that aims to capture health impacts of illness and benefits of treatments across the whole spectrum of physical and mental health. One of its key strengths is that at just 5 questions long, completion rates are typically high. Another is that our findings can inform future cost-effectiveness analyses. However, a drawback of the EQ-5D is that it may be less sensitive to some health impacts than others. For example, some of the health domains (e.g. mobility, self-care) may be largely unaffected by the death of one’s baby and our data support this. Yet, we have still shown a clear and significant utility shortfall in parents whose baby died. This reflects the findings from mental health research where the EQ-5D has been shown to capture the impact of mental illness on people’s health [24, 25]. To maximise the potential responsiveness of the EQ-5D in our study, we used the 5-level version (rather than the 3-level version). The 3-level version has a possible 243 health profiles (i.e. a score of 1, 2, or 3 on each of 5 domains), whereas the 5-level version has 3125 possible health profiles (i.e. a score of 1, 2, 3, 4, or 5 on each of 5 domains).

### Implications and future research

An important use of our findings is to produce more robust cost-effectiveness evidence for interventions in maternity care. In particular, those aiming to support bereaved parents as we now have an estimate of their utility shortfall. This can be used in both *ex ante* and *ex post* cost-effectiveness models where the aim is to estimate an incremental cost-effectiveness ratio in terms of cost per QALY. As this is the first published estimate of this kind, our findings may also be relevant and useful in other countries where they use a cost-effectiveness threshold in terms of “cost per QALY” [2]. However, it should be noted that decision-making bodies may also be interested in broader measures of cost-effectiveness rather than cost–utility analyses alone, especially in the context of complex interventions, which those relating to baby death are likely to be. For example, public sector (non-healthcare) organisations that are funding preventative/public health interventions may prefer to consider health benefits in monetary terms in the format of a cost–benefit analysis or be interested in what the monetary return on their investment may be. However, there are important ethical considerations for trying to attach a monetary value to a baby’s life and so this approach may have significant challenges in this context. Another alternative is to report QALYs as one outcome as part of a cost–consequence framework that considers and reports disaggregated health and non-health impacts from the perspective of different sectors [26].

Better understanding of the complexity of the impact of subsequent pregnancy is an important area for future research, and our findings here should be interpreted with the caveat that only 68 parents in our sample had not had a subsequent pregnancy. Our study included mothers/birthing parents and fathers/partners. In other studies, those in the latter group report experiencing specific challenges and health impacts following the death of their baby [27]. Fathers/partners often do not disclose their own needs, or have their own needs met as they feel the need to remain emotionally strong for their partner [28]. The utility shortfall for fathers/ partners in our sample was approximately 6%, and utility values were not significantly different from the general population. Another area for further focussed research is fathers/partners as there were fewer than 20 participants in this subgroup of our sample.

It is also important to explore the impact of baby death on parents in representative samples and alternative outcome measures. For example, more than 12% of parents who have experienced perinatal loss have post-traumatic stress disorder in the years following their loss [29]. It may be possible to use linked electronic health records alongside representative population samples (e.g. The Household Survey for England) to systematically identify bereaved parents and learn more about their health and wellbeing. Another method to increase representativeness could be to use birth cohort studies that recruit at the time of pregnancy and collect longitudinal information on parents’ health and quality of life, including those whose baby dies.

## Conclusions

There is a significant and long-lasting impact of perinatal death on parents’ health, driven largely by symptoms of anxiety and/or depression. It is vital that parents are given appropriate and tailored support following perinatal death. More research is required to determine the most effective and cost-effective means of providing psychological support following perinatal bereavement.

## Supplementary Information

Below is the link to the electronic supplementary material.

Supplementary file1 (DOCX 18 KB)

## Data Availability

The data generated and analysed during the current study are available from the corresponding author upon reasonable request.
