# Supplementary material for: Measuring EQ-5D-5L utility values in parents who have experienced perinatal death
Source: Eur J Health Econ. 2024 Feb 25;25(8):1383–91. doi: 10.1007/s10198-024-01677-z (PMC11442506; doi:10.1007/s10198-024-01677-z)
Supplement: Supplementary file 1 — Supplementary file1 (DOCX 18 KB) [file 10198_2024_1677_MOESM1_ESM.docx]

**Measuring EQ-5D-5L utility values in parents who have experienced perinatal death**

**Box 1.** Calculating quality adjusted life years (QALYs)

| ***Calculating quality adjusted life years (QALYs)***  Health utility is measured on a 0-1 scale. QALYs are calculated by multiplying differences in utility values (e.g. a utility shortfall) by the duration of the time period (measured in years) over which they are experienced. The table below provides some examples in the context of utility shortfalls compared to the general population.   \| Health utility of general population \| Health utility of comparator sample \| Utility shortfall in comparator sample \| Number of years with shortfall \| QALY loss \| \| --- \| --- \| --- \| --- \| --- \| \| 0.90 \| 0.60 \| 0.3 \| 1 \| 0.3 \| \| 0.85 \| 0.60 \| 0.25 \| 5 \| 1.25 \| \| 0.80 \| 0.70 \| 0.1 \| 2 \| 0.2 \| \| 0.75 \| 0.70 \| 0.05 \| 3 \| 0.15 \| |
| --- | --- | --- | --- | --- | --- | --- | --- | --- | --- | --- | --- | --- | --- | --- | --- | --- | --- | --- | --- | --- | --- | --- | --- | --- | --- |

**Table S1.** Utility values for women in the general population sample used as comparator in the current study and mothers/birthing parents in our sample, by age

| Age (years) | Mean general population utility value (based on Hernandez et al*) | Mean utility in study sample |
| --- | --- | --- |
| 18-34 | 0.909 | 0.747 |
| 35-44 | 0.886 | 0.780 |
| 45-54 | 0.859 | 0.808 |
| 55+ | 0.812 | 0.745 |

*Hernandez Alava, M, Pudney, S, Wailoo, A: Estimating EQ-5D by Age and Sex for the UK. NICE Decision Support Unit (DSU), Sheffield (2022)
